# Expert Evidence

**Published:** 1899-12-23

**Authors:** 


### EXPERT EVIDENCE.

We have long been accustomed to hear nasty things said about conflicting medical evidence in courts of law, and endless have been the suggestions which have been made to render expert evidence more satisfactory. In view, then, of the many hard things that have been said from time to time about expert medical evidence, and about expert evidence in general, it is not without interest to refer to a paper read at a recent meeting of the New York State Medical Association by Mr. Purrington,[1] a counsellor-at-law. He takes up the question, What is meant by an expert? to which he answers that an expert is only a person specially experienced, skilled, or learned in some art or science wherein the formation of a sound opinion necessitates a previous course of study and experience beyond the lines followed by the average man. Then he goes on to point out that lawyers are to be regarded as experts in matters of law, and as it is from lawyers that some of the strongest strictures come upon the unreliability of medical expert evidence, it is, he suggests, worth consideration how far the very highest experts in law are able to arrive at unanimity of utterance upon the simple questions of law which are from time to time submitted to them. "You laymen," he says, "would think for instance that it is much easier to define the word malice, to say whether a corporation is liable for its servants' malicious acts, whether certain words are defamatory within the ordinary understanding of reasonable men, and whether a criminal statute is retro-active in effect and, therefore, *ex post facto*, than it is to tell whether certain physical symptoms indicate typhoid fever or appendicitis, whether morphine poisoning can be absolutely diagnosticated from symptoms alone, or what, with reasonable certainty is the prognosis of a morbid condition; say, traumatic hysteria. Let us see. Since memory runs not to the contrary, English judges have cudgelled their brains over the legal definition of malice; and yet only recently in the great case of Allen *v.* Flood, involving that definition, two judges and six law lords reversed the judgment of ten judges and three law lords." He then goes on to give detailed instances of the reversal of judgments by the several Courts as showing divergence in what is to be regarded as the very highest type of legal expert evidence, and, coming across to the American side of the water, he cites a case in which the facts were agreed upon, and the only question involved was one of law. "The trial judge decided negatively. The appellate division of this department, by a vote of three to two, reversed him; the Court of Appeals in turn reversed the appellate division, two of its judges dissenting; and finally, the Supreme Court of the United States, after two arguments, affirmed the Court of Appeals, three of its members dissenting. Surely no one would presume, because of these differences of opinion, to doubt for a moment the ability, the learning, the absolute honesty, of these various experts of the law; and it is equally manifest that the frequent differences of opinion among medical experts—the only class we are now considering—should not of themselves alone justify the criticism so freely made." It has been said from the Bench that "it is generally safer to take the judgments of unskilled jurors than the opinions of hired and generally biassed experts," and many of us have perhaps smiled at the cynical classification of liars in which the first place is given to experts; but while, no doubt, instances may be met with here and there in which expert evidence, just the same as any other form of evidence, may prove unreliable, that is no reason why in the one case any more than in the other reflections should be cast upon a whole class of witnesses. "I have sometimes wondered," says the author of the paper, "whether those judges who have been most denunciatory of the differing conclusions at which reputable medical experts have arrived at upon the facts in evidence, have reflected that they, too, belong to a body of experts in the law, whose members often differ, very honestly and ably, in drawing conclusions, even upon agreed facts."

[1] New York Med. Rec., Dec. 9.

